# Severe Hyperandrogenism Revealing a 46,XY Disorder of Sex Development Due to a Novel NR5A1 Mutation

**DOI:** 10.7759/cureus.106963

**Published:** 2026-04-13

**Authors:** Fatimazahra Yakine, Ilham Bouarab, Fatima Zahra Alaoui-Inboui, Fadoua Idrissifawzi, Bouchra Slaoui

**Affiliations:** 1 Paediatric Endocrinology and Diabetology Unit, Department of Paediatrics 2, Abderrahim Harouchi Mother and Children University Hospital, Casablanca, MAR; 2 Paediatric Pneumology and Allergology Unit, Department of Paediatrics 2, Abderrahim Harouchi Mother and Children University Hospital, Casablanca, MAR; 3 Department of Paediatric Surgery, Abderrahim Harouchi Mother and Children University Hospital, Casablanca, MAR

**Keywords:** 46xy dsd, disorders of sex development, hirsutism, hyperandrogenism, nr5a1, steroidogenic factor 1

## Abstract

46,XY disorders of sex development (DSDs) are rare congenital conditions resulting from impaired gonadal differentiation and/or steroidogenesis. The *NR5A1 *gene encodes steroidogenic factor 1 (SF-1), a key regulator of gonadal development and endocrine function, and pathogenic variants are associated with a broad phenotypic spectrum that may present beyond the neonatal period. We report the case of a 12-year-and-6-month-old phenotypic female adolescent presenting with severe hirsutism, acne, primary amenorrhea, and progressive virilization. Hormonal evaluation demonstrated hyperandrogenism with hypergonadotropic hypogonadism. Imaging revealed the absence of a normal uterus and ovaries, with the presence of a Müllerian remnant, along with bilateral gonadal structures consistent with testicular tissue, and cytogenetic analysis showed a 46,XY karyotype. Genetic testing identified a novel heterozygous pathogenic *NR5A1* splice-site variant (c.871-2A>G), confirming the diagnosis of 46,XY DSD. Multidisciplinary management included bilateral gonadectomy followed by estrogen replacement therapy. This case highlights an uncommon adolescent presentation of 46,XY DSD revealed by severe hyperandrogenism in a phenotypic female individual and underscores the importance of molecular genetic testing for accurate diagnosis, management, and long-term follow-up.

## Introduction

Disorders of sex development (DSDs) are congenital conditions arising from incongruence among chromosomal, gonadal, and phenotypic sex, with an estimated prevalence of approximately one in 5,000 live births (0.05%) [1].

According to the Chicago Consensus Conference (2006), updated in 2016, DSDs are classified into three main categories: 46,XY DSD, 46,XX DSD, and DSDs associated with sex chromosome abnormalities [2]. The global incidence of 46,XY DSD is estimated at approximately one in 20,000 live births [1].

The *NR5A1* gene (nuclear receptor subfamily 5 group A member 1), located on chromosome 9, encodes steroidogenic factor 1 (SF-1), a transcription factor essential for steroidogenesis and for the development and function of the hypothalamic-pituitary-gonadal and adrenal axes [3]. Pathogenic variants in *NR5A1* represent one of the most common genetic causes of 46,XY DSD, accounting for approximately 10-20% of cases [4].

The phenotypic spectrum associated with *NR5A1* mutations is highly variable, ranging from isolated hypospadias to complete testicular dysgenesis and infertility in 46,XY individuals, as well as primary ovarian insufficiency or ovotesticular DSD in 46,XX individuals. To date, no clear and consistent genotype-phenotype correlation has been established [5].

Adolescent presentation of *NR5A1*-related DSD is uncommon but well documented, particularly in cases presenting with hyperandrogenism, such as severe hirsutism. Although adrenal insufficiency is rare in heterozygous *NR5A1* mutations [6], extragonadal anomalies, including splenic abnormalities and other congenital malformations, have been reported [7]. Management requires a multidisciplinary approach and long-term endocrine, psychological, and reproductive follow-up.

The objective of this case report was to describe the clinical, biochemical, genetic, and therapeutic features of an adolescent girl diagnosed with 46,XY DSD.

We report the case of a 12-year-and-6-month-old phenotypic female adolescent who presented with hirsutism and acne. Endocrine evaluation revealed a 46,XY DSD associated with a heterozygous mutation in the *NR5A1* gene.

## Case presentation

A 12-year-and-6-month-old phenotypic female adolescent, born to parents reported as non-consanguineous based on family history, was referred for evaluation of progressive virilization. There was no family history of pubertal disorders, infertility, DSD, or unexplained childhood deaths. The patient was not receiving chronic medication, including corticosteroids. Symptoms began at 11 years and two months of age with the onset of acne and hirsutism. The acne was unresponsive to topical retinoids. Over time, hirsutism worsened and was accompanied by voice deepening, prompting evaluation for hyperandrogenism.

**Figure 1:**
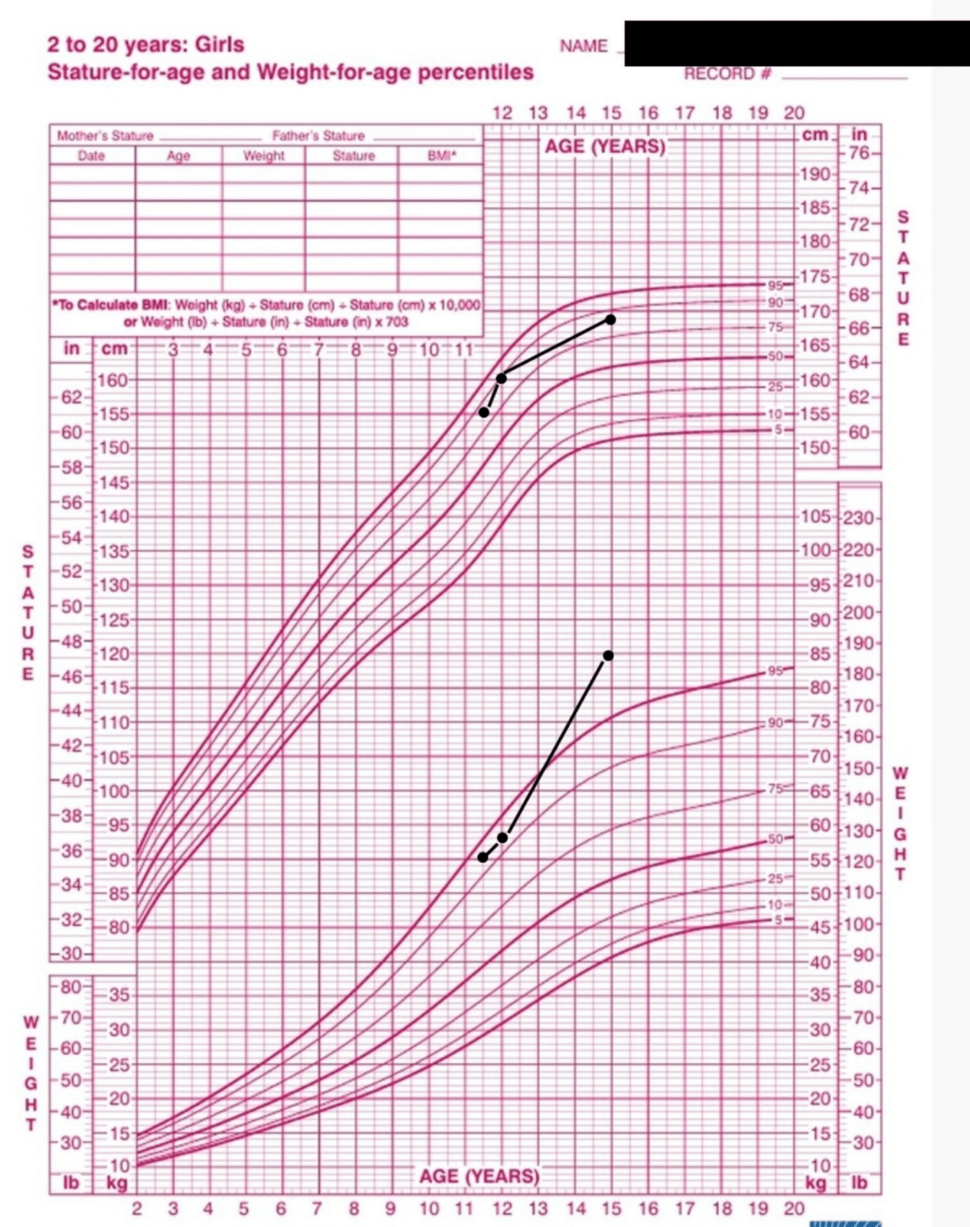
Growth parameters of the patient. Height and weight growth curves showing accelerated growth velocity during follow-up.

Physical examination revealed a female phenotype with severe hirsutism characterized by coarse, pigmented terminal hair involving the face, upper and lower limbs, and abdomen. The modified Ferriman-Gallwey score was 26, indicating severe hirsutism (a standardized scoring system assessing androgen-dependent hair growth). The patient had not yet attained menarche, without galactorrhea or clinical signs of hypercortisolism. Pubertal development corresponded to Tanner stage B3-P3. Genital examination revealed mild clitoromegaly (clitoral length: 12 mm), two separate perineal openings with a non-blind-ending vaginal orifice, and posterior labial fusion. No gonads were palpable. The anogenital distance was within the normal female range. Growth parameters showed a height of 164 cm (+1.5 SD), weight of 75 kg (>+2 SD), and body mass index of 28.2 kg/m², consistent with overweight, based on female reference growth charts (Figure 1) [8].

Hormonal evaluation

Initial hormonal assessment showed normal levels of 17-hydroxyprogesterone, morning serum cortisol, and plasma adrenocorticotropic hormone, excluding an adrenal origin. Gonadotropin levels were markedly elevated with suppressed estradiol levels, consistent with hypergonadotropic hypogonadism. Total testosterone was elevated compared to female reference ranges and was inappropriately high for age. Anti-Müllerian hormone was very low, and inhibin B was undetectable. This biochemical profile was suggestive of gonadal dysfunction, although the exact origin of androgen excess could not be definitively determined. Detailed laboratory results are summarized in Table 1.

**Table 1:** Initial hormonal laboratory assessment and corresponding reference ranges. Reference ranges correspond to female laboratory standards for age. Units are expressed in conventional laboratory units. ACTH, adrenocorticotropic hormone; FSH, follicle-stimulating hormone; LH, luteinizing hormone; AMH, anti-Müllerian hormone.

Parameter	Result	Reference range (female)
17-hydroxyprogesterone (ng/mL)	1.20	0.1-2.0
Morning serum cortisol (µg/L)	110.20	50-250
ACTH (plasma) (pg/mL)	32.35	10-60
FSH (mIU/mL)	56.91	1-10
LH (mIU/mL)	17.67	1-8
Estradiol (pg/mL)	<24	30-400
Total testosterone (ng/mL)	2.36	0.1-0.5
AMH (ng/mL)	0.28	0.96-13.34
Inhibin B (pg/mL)	Undetectable	40-200

Radiological findings

Abdominopelvic ultrasound demonstrated a complete absence of the uterus and ovaries. Bilateral oval, heterogeneous echogenic structures were identified in the inguinal regions, consistent with gonadal tissue of testicular appearance. The right gonadal structure measured 2.2 × 0.8 × 1.2 cm (estimated volume: 1.34 mL), and the left measured 2.3 × 0.8 × 1.3 cm (estimated volume: 1.5 mL), both markedly reduced in size for age, suggesting dysgenetic testes.

**Figure 2:**
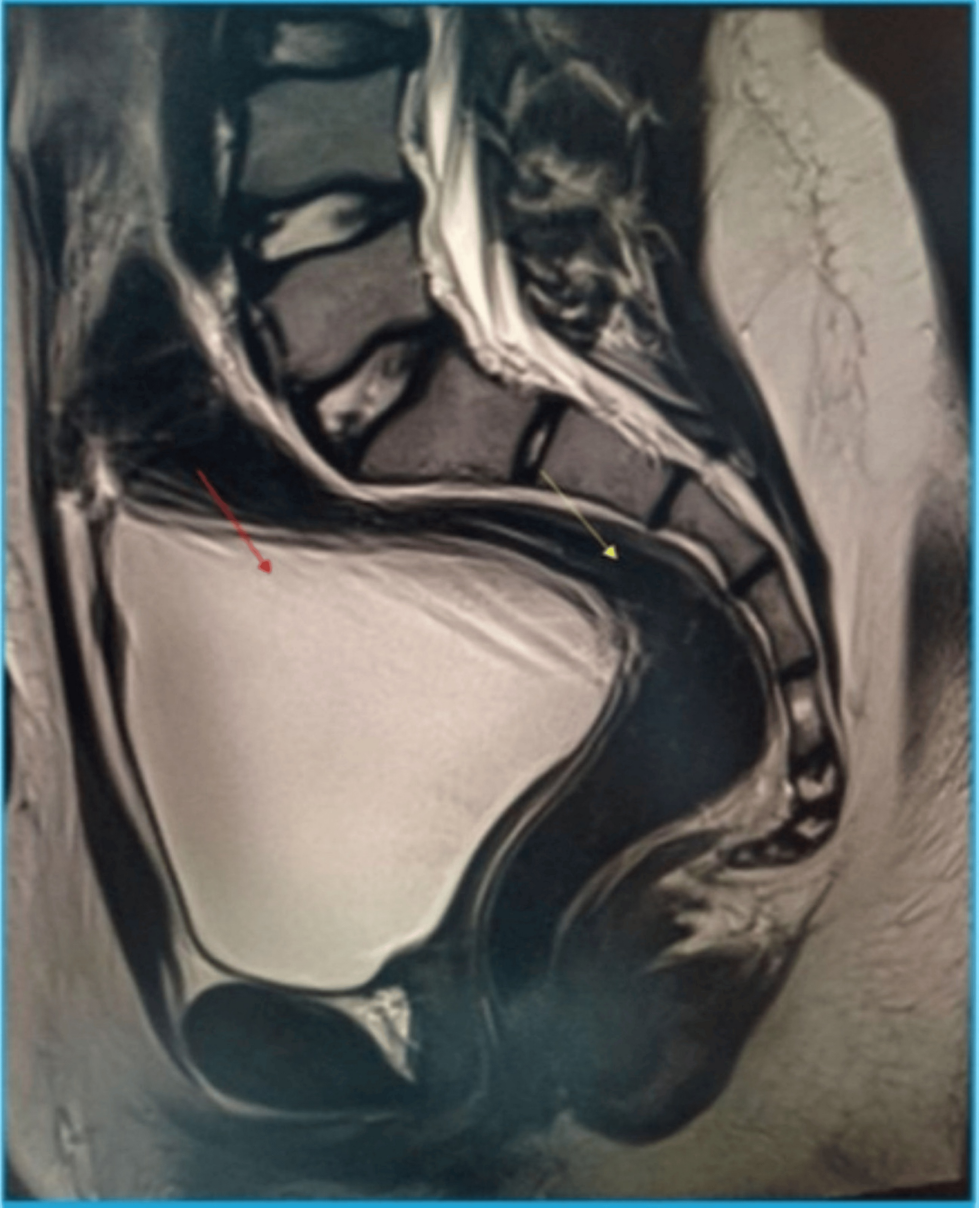
Abdominopelvic MRI. Sagittal MRI section demonstrating absence of the uterus. The urinary bladder (red arrow) is seen anteriorly, and the rectum (yellow arrow) posteriorly. MRI, magnetic resonance imaging.

**Figure 3:**
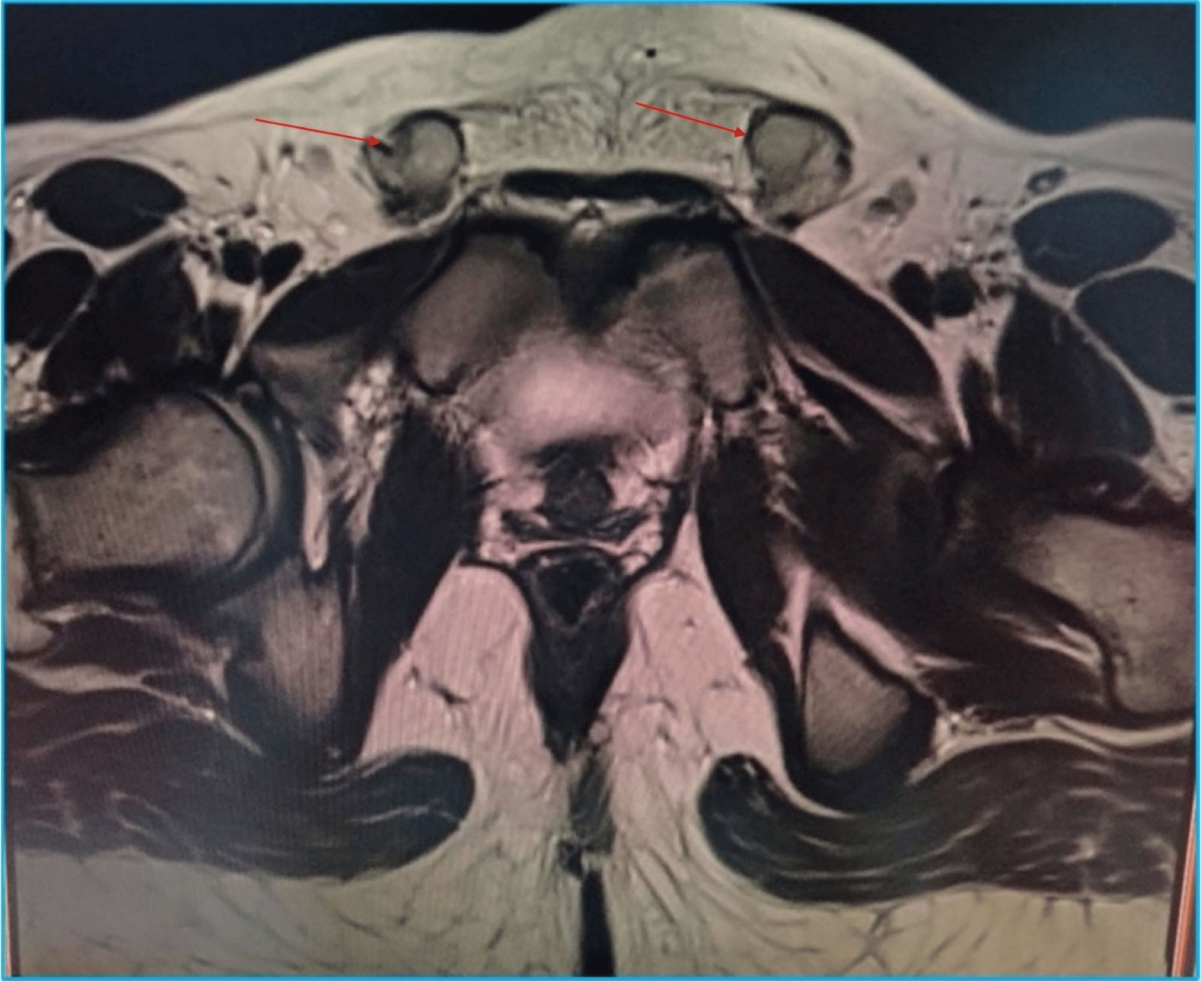
Abdominopelvic MRI. Axial MRI section showing bilateral inguinal gonadal tissue (red arrow). MRI, magnetic resonance imaging.

Abdominopelvic magnetic resonance imaging (MRI) demonstrated a small intervesicorectal structure deviated to the right, measuring approximately 22 mm in its greatest dimension, compatible with a Müllerian remnant. In addition, bilateral small gonadal structures were visualized in the inguinal regions, consistent with dysgenetic testicular tissue. No ovarian parenchyma was identified. Overall, the radiological findings supported a diagnosis of gonadal-origin 46,XY DSD with dysgenetic testes and incomplete Müllerian regression (Figures 2, 3).

Genetic analysis

Cytogenetic analysis revealed a 46,XY karyotype, confirming discordance between chromosomal and phenotypic sex. Targeted DNA sequencing identified a novel heterozygous pathogenic variant in the *NR5A1* gene, c.871-2A>G, affecting a splice acceptor site. This variant had not been previously reported and was classified as pathogenic according to American College of Medical Genetics and Genomics (ACMG) criteria, supporting a diagnosis of 46,XY DSD related to *NR5A1* mutation.

Surgical and histopathological findings

**Figure 4:**
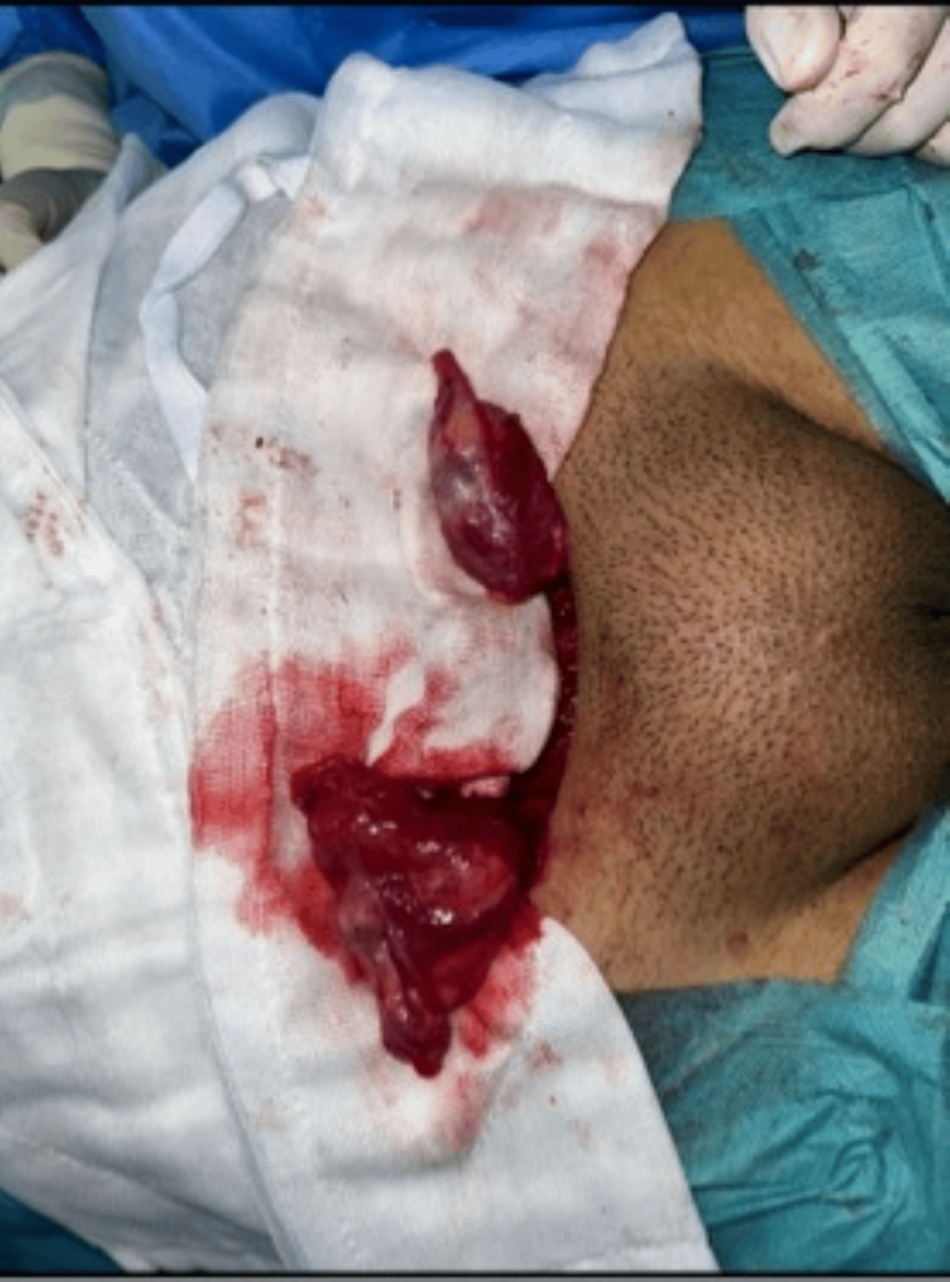
Intraoperative appearance of bilateral gonadal structures. Surgical exploration demonstrating dysgenetic gonadal tissue located in the inguinal region.

Bilateral gonadal exploration with biopsy was performed. Both gonads were oval, whitish, and small, located inguinally, without an identifiable epididymis or vas deferens. Tubal-like structures were present bilaterally, without uterine tissue or peritoneal extension (Figure 4).

Histopathological examination revealed dysgenetic testicular tissue with a limited number of medium-sized seminiferous tubules showing maturation arrest at the primary spermatocyte stage, sparse Leydig cells, and absence of ovarian parenchyma or stroma. These findings were consistent with dysgenetic testicular tissue.

Management and follow-up

Management was discussed in a multidisciplinary setting involving pediatric endocrinologists, pediatric surgeons, geneticists, and psychologists. Age-appropriate information was provided, and shared decision-making was conducted with the patient and her parents. The patient expressed a stable female gender identity. Bilateral orchiectomy was performed in accordance with the declared gender, with psychological support. Histological analysis of the resected gonads showed complete testicular atrophy without intratubular germ cell neoplasia or malignant features. Estrogen replacement therapy with 17β-estradiol was subsequently initiated, along with management of hyperandrogenism and regular endocrinological follow-up.

## Discussion

46,XY DSDs represent a heterogeneous group of conditions resulting from impaired gonadal differentiation and/or steroidogenesis, with marked phenotypic variability. Among the genes involved, *NR5A1* (SF-1) plays a central role due to its critical function in gonadal development and regulation of multiple steroidogenic enzymes at the intersection of the hypothalamic-pituitary-gonadal and adrenal axes. Pathogenic variants in *NR5A1* are increasingly recognized as a relatively frequent cause of 46,XY DSD, with increasing detection attributable to the widespread use of next-generation sequencing technologies [9].

Classically, 46,XY DSD is identified in the neonatal period because of genital ambiguity. However, *NR5A1* mutations are associated with a particularly broad clinical spectrum, including hypospadias, partial or complete testicular dysgenesis, male infertility, and phenotypic female presentations diagnosed later in adolescence, often in the context of primary amenorrhea or progressive hyperandrogenism manifested by severe hirsutism, acne, and voice deepening. These presentations may occur in the absence of significant genital ambiguity at birth [10]. Delayed presentation is likely related to dysgenetic and often fluctuating testicular function, which may sustain sufficient androgen production to induce secondary virilization, while Sertoli cell function is frequently impaired, as reflected by low anti-Müllerian hormone and inhibin B levels. Internal genital tract differentiation depends on the initial embryological environment and the degree of gonadal dysgenesis [11]. In the present case, the association of elevated testosterone levels, hypergonadotropic hypogonadism, suppressed anti-Müllerian hormone, and the absence of normal uterine and ovarian structures with visualization of structures consistent with testicular tissue strongly supports a diagnosis of gonadal-origin 46,XY DSD, in line with phenotypes described in cohorts of patients with NR5A1 mutations [11]. Presentation with severe hirsutism represents an uncommon but well-documented phenotype in the literature.

Although *NR5A1* is involved in adrenal development and function, primary adrenal insufficiency remains rare in heterozygous forms. Nevertheless, systematic evaluation through clinical assessment and biochemical testing, including cortisol and adrenocorticotropic hormone measurements, is recommended, particularly in the presence of suggestive features, warranting targeted follow-up [9]. This approach is consistent with the present case, in which adrenal function remained normal throughout follow-up.

Beyond gonadal abnormalities, *NR5A1* mutations have been associated with a wide range of extragonadal manifestations, including splenic abnormalities, various endocrine disorders (hypogonadism, cryptorchidism, bilateral anorchia, ovarian insufficiency), infertility, adrenal masses, and gynecological conditions such as polycystic ovary syndrome and endometriosis. Neurological, skeletal, and cardiovascular manifestations have also been reported. This phenotypic diversity reflects the broad expression of *NR5A1* in steroidogenic and non-steroidogenic tissues, including the brain, spleen, and placenta [12,13]. These findings underscore the importance of comprehensive phenotypic evaluation extending beyond gonadal and genital features alone [14].

To date, more than 80 distinct *NR5A1* variants distributed throughout the protein have been described. The absence of a strict genotype-phenotype correlation complicates clinical interpretation and underscores the value of systematic molecular genetic testing in DSD. Identical variants may be associated with markedly different phenotypes, even within the same family, suggesting the influence of modifier genes or environmental factors [14]. To our knowledge, this splice-site variant has not been previously reported, further expanding the mutational spectrum of *NR5A1*-associated DSDs and supporting the growing genetic heterogeneity of this condition.

In 46,XY individuals with dysgenetic gonads containing Y-chromosomal material, the risk of gonadal tumors, particularly gonadoblastoma and germ cell tumors, represents a major clinical concern. Current international recommendations advocate an individualized approach based on phenotypic characteristics, gonadal location, estimated tumor risk, patient and family preferences, and the potential for preservation of endogenous hormonal function. Updated international DSD consensus guidelines emphasize the necessity of a multidisciplinary approach and age-appropriate information [15].

In the present case, multidisciplinary discussion, consideration of a stable female gender identity, and histological findings consistent with dysgenetic testicular tissue without evidence of malignancy supported the chosen therapeutic strategy. Subsequent initiation of estrogen replacement therapy with 17β-estradiol addressed goals related to female pubertal development, bone health, and psychosocial well-being.

Finally, this case highlights that severe hirsutism in a phenotypic female adolescent, particularly when associated with progressive virilization, delayed menarche, and/or abnormalities of the gonadotropic axis, should raise suspicion of an underlying DSD. Important differential diagnoses include polycystic ovary syndrome, non-classical congenital adrenal hyperplasia, androgen-secreting tumors, and aromatase deficiency. This case also underscores the value of genetic testing in establishing the diagnosis, reducing diagnostic delay, guiding multidisciplinary management, and anticipating long-term endocrine, oncological, psychological, and reproductive outcomes.

## Conclusions

This case highlights the marked phenotypic variability of 46,XY DSD related to *NR5A1* mutations and illustrates an uncommon adolescent presentation revealed by severe hyperandrogenism in a phenotypic female individual. Consideration of a DSD etiology is essential in adolescents presenting with severe hirsutism, progressive virilization, and primary amenorrhea, even in the absence of neonatal genital ambiguity.

Molecular genetic diagnosis was pivotal in confirming the etiology, guiding therapeutic decisions, and supporting multidisciplinary management respectful of gender identity. This case underscores the importance of systematic genetic evaluation and long-term follow-up in late-presenting DSD.

## References

[1] Ding L, Luo M, Deng S, Zhang D, Tian Q (2025). The clinical diversity and molecular etiology in 46, XY disorders of sex development patients without uterus. Orphanet J Rare Dis.

[2] Vicario S, Escolino M, Esposito G, Porcaro M, Di Mase R, Azizoglu M, Esposito C (2025). Clinical spectrum, surgical management, and outcomes of NR5A1-related 46,XY differences of sex development: A narrative review. Medicina (Kaunas).

[3] Wei X, Li S, He Y (2023). NR5A1-related 46,XY partial gonadal dysgenesis: A case report and literature review. Medicine (Baltimore).

[4] Alhamoudi KM, Alghamdi B, Aljomaiah A, Alswailem M, Al-Hindi H, Alzahrani AS (2022). Case report: Severe gonadal dysgenesis causing 46,XY disorder of sex development due to a novel NR5A1 variant. Front Genet.

[5] Domenice S, Machado AZ, Ferreira FM (2016). Wide spectrum of NR5A1-related phenotypes in 46,XY and 46,XX individuals. Birth Defects Res C Embryo Today.

[6] Kumar S, Pandit R, Sarathi V (2025). 46, XY under-virilization and NR5A1 variants: Monocentric Indian experience and systematic review. Ann Endocrinol (Paris).

[7] Gialouris JV, Cheong PL, Zekanovic S, Wang M, Wijewardene A (2025). Two cases of 46,XY differences of sex development due to gonadal dysgenesis associated with novel NR5A1 variants. JCEM Case Rep.

[8] (2026). CDC Growth Charts: Data Files. https://www.cdc.gov/growthcharts/cdc-data-files.htm.

[9] Güneş S, Sevim RD, Yiğit ZM, Çulhacı N, Ünüvar T, Anık A (2023). Pubertal virilization in an adolescent with 46, XY disorder of sexual development: A novel mutation in NR5A1 gene. Acta Endocrinol (Buchar).

[10] Kostopoulou E, Eliades A, Papatheodoropoulou A (2025). 46,ΧΥ DSD in an adolescent with a novel de novo variant of the NR5A1 gene - Case report and literature review. Hormones (Athens).

[11] Lee PA, Nordenström A, Houk CP (2016). Global disorders of sex development update since 2006: Perceptions, approach and care. Horm Res Paediatr.

[12] Luppino G, Wasniewska M, Coco R (2024). Role of NR5A1 gene mutations in disorders of sex development: Molecular and clinical features. Curr Issues Mol Biol.

[13] Kouri C, Sommer G, Martinez de Lapiscina I (2024). Clinical and genetic characteristics of a large international cohort of individuals with rare NR5A1/SF-1 variants of sex development. EBioMedicine.

[14] Sasaki T, Suzuki S, Ono M (2024). Case report: Rare heterozygous variant in the NR5A1 gene causing 46,XY complete gonadal dysgenesis with a non-communicating rudimentary uterus. Front Med (Lausanne).

[15] Lu L, Luo F, Wang X (2022). Gonadal tumor risk in pediatric and adolescent phenotypic females with disorders of sex development and Y chromosomal constitution with different genetic etiologies. Front Pediatr.

